# Minimal Residual Disease in Chronic Lymphocytic Leukemia: A New Goal?

**DOI:** 10.3389/fonc.2019.00689

**Published:** 2019-08-29

**Authors:** Ilaria Del Giudice, Sara Raponi, Irene Della Starza, Maria Stefania De Propris, Marzia Cavalli, Lucia Anna De Novi, Luca Vincenzo Cappelli, Caterina Ilari, Luciana Cafforio, Anna Guarini, Robin Foà

**Affiliations:** ^1^Hematology, Department of Translational and Precision Medicine, Sapienza University of Rome, Rome, Italy; ^2^GIMEMA Foundation, Rome, Italy; ^3^Department of Molecular Medicine, Sapienza University of Rome, Rome, Italy

**Keywords:** chronic lymphocytic leukemia, minimal residual disease, flow cytometry, droplet digital PCR, next generation sequencing, ibrutinib, venetoclax

## Abstract

In chronic lymphocytic leukemia (CLL), there is a growing interest for minimal residual disease (MRD) monitoring, due to the availability of drug combinations capable of unprecedented complete clinical responses. The standardized and most commonly applied methods to assess MRD in CLL are based on flow cytometry (FCM) and, to a lesser extent, real-time quantitative PCR (RQ-PCR) with allele-specific oligonucleotide (ASO) primers of immunoglobulin heavy chain genes (IgH). Promising results are being obtained using droplet digital PCR (ddPCR) and next generation sequencing (NGS)-based approaches, with some advantages and a potential higher sensitivity compared to the standardized methodologies. Plasma cell-free DNA can also be explored as a more precise measure of residual disease from all different compartments, including the lymph nodes. From a clinical point of view, CLL MRD quantification has proven an independent prognostic marker of progression-free survival (PFS) and overall survival (OS) after chemoimmunotherapy as well as after allogeneic transplantation. In the era of mechanism-driven drugs, the paradigms of CLL treatment are being revolutionized, challenging the use of chemoimmunotherapy even in first-line. The continuous administration of ibrutinib single agent has led to prolonged PFS and OS in relapsed/refractory and treatment naïve CLL, including those with *TP53* deletion/mutation or unmutated *IGHV* genes, though the clinical responses are rarely complete. More recently, chemo-free combinations of venetoclax+rituximab, venetoclax+obinutuzumab or ibrutinib+venetoclax have been shown capable of inducing undetectable MRD in the bone marrow, opening the way to protocols exploring a MRD-based duration of treatment, aiming at disease eradication. Thus, beside a durable disease control desirable particularly for older patients and/or for those with comorbidities, a MRD-negative complete remission is becoming a realistic prospect for CLL patients in an attempt to obtain a long-lasting eradication and possibly cure of the disease. Here we discuss the standardized and innovative technical approaches for MRD detection in CLL, the clinical impact of MRD monitoring in chemoimmunotherapy and chemo-free trials and the future clinical implications of MRD monitoring in CLL patients outside of clinical trials.

## Introduction

Chronic lymphocytic leukemia (CLL), characterized by the clonal expansion of mature B lymphocytes in the peripheral blood (PB), bone marrow (BM), spleen and lymph nodes (1), is diagnosed in the presence of at least 5,000 circulating clonal B lymphocytes per microliter sustained for at least 3 months, with a distinctive morphology and a typical immunophenotype (2, 3). CLL represents the most common leukemia in the Western world, predominates in the elderly and its incidence increases exponentially with age (4). The clinical outcome of CLL patients is extremely variable: beside cases with aggressive disease at onset often requiring immediate treatment, there are patients with an initial indolent phase followed by disease progression and others who do not progress for decades or ever. This is largely explained by the heterogeneity of the biologic features, some with well-known prognostic implications. Somatic mutations of the immunoglobulin heavy chain variable (*IGHV*) genes, CD49d and CD38 expression, along with the identification of chromosomal abnormalities (deletions of chromosome 13q, 17p, and 11q, and trisomy 12) and of recurrent mutations in *TP53, ATM, NOTCH1, BIRC3*, and *SF3B1* genes, have all been recognized as features capable of predicting the outcome of CLL patients (5–9).

Patients are treated only when the disease becomes symptomatic and/or progresses to more advanced clinical stages. Effective treatments include combinations of chemotherapy (fludarabine, cyclophosfamide (FC); bendamustine (B); chlorambucil (Chl) with anti-CD20 monoclonal antibodies (rituximab (R), obinutuzumab (GA-101, G) or novel agents such as the B-cell receptor (BCR) inhibitors (anti-BTK ibrutinib and acalabrutinib; anti-PI3K idelalisib and duvelisib) or BCL2 inhibitors (venetoclax), introduced in the last 5 years. Treatment choice is currently based on patients' age and comorbidities, disease biology (*TP53* deletion/mutation and *IGHV* mutations) and status [treatment naïve (TN), relapsed/refractory (R/R)], but international guidelines are rapidly changing the current paradigms thanks to the efficacy of novel drugs/drug combinations (see below).

Unlike in adult and childhood acute lymphoblastic leukemia (ALL), where the assessment of response to therapy by minimal residual disease (MRD) monitoring drives therapeutic choices, MRD analysis in CLL has been only recently introduced.

Whilst morphologic response criteria are not sufficiently sensitive to predict outcome after treatment, several studies have demonstrated that patients who achieved a clinical complete remission (CR) as defined in the International Workshop on CLL (iwCLL) response criteria (2) but with residual CLL cells can experience a disease relapse due to the expansion of the latter (10–12). Contrariwise, an undetectable MRD (uMRD) by highly sensitive techniques identifies CLL patients with a prolonged progression-free survival (PFS) and overall survival (OS) irrespective of the clinical response (CR or partial response, PR) in the context of chemoimmunotherapy regimens (13–16).

As a consequence, MRD analysis has been recently approved as an intermediate/surrogate endpoint to assess treatment efficacy in randomized clinical trials designed to show a superiority in terms of PFS in CLL patients by the European Medicines Agency (EMA) (EMA guidelines), with patients who achieve clinical CR and uMRD (<10^−4^) being considered MRD responders. However, the predicted benefit on PFS needs to be confirmed with longer follow-up (17). Along this line, the most recent iwCLL guidelines recommend MRD assessment with standardized methods in CLL patients enrolled in clinical trials aiming at obtaining a deep response (2). Furthermore, MRD monitoring in the setting of stem cell transplant (SCT) procedures has proven effective to evaluate disease kinetics after transplant (18, 19). Currently, with the introduction of novel agents targeting the BCR or the BCL2 protein in the therapeutic armamentarium of CLL, the clinical significance of MRD has been reassessed. Some of the novel therapies, i.e., ibrutinib and idelalisib plus rituximab, induce in fact a prolonged control of the disease and survival with only very few patients achieving a CR and MRD is therefore not applicable (20, 21). On the other hand, venetoclax-based regimens can induce a high proportion of uMRD both in the PB and BM, even in advanced lines of therapy (see below) (22, 23).

The common and standardized methods for MRD monitoring are represented by flow cytometry (FCM) and real-time quantitative polymerase chain reaction (RQ-PCR) (24–28). More recently, promising results have been obtained by droplet digital PCR (ddPCR) and next generation sequencing (NGS)-based approaches (29–34).

In this review, we discuss the standardized and innovative technical approaches for MRD detection in CLL, the clinical impact of MRD monitoring in chemoimmunotherapy and chemo-free trials, and the future clinical implications of MRD monitoring in CLL patients outside of clinical trials.

## Techniques To Study MRD

The general principles to be fulfilled for a MRD detection assay are the capability of identifying malignant cells on the basis of a univocal profile that is not shared by their normal counterparts and the reliability and reproducibility of the method, that should be easy to perform and simple to interpret. The two major approaches satisfying these criteria are FCM and allele-specific oligonucleotide (ASO) of immunoglobulin heavy chain (IgH) RQ-PCR. The availability of standardized guidelines both for FCM (24–26) and ASO IgH RQ-PCR (34) allows to accurately define the amount of residual cells in CLL samples with inter-laboratory comparability, with a minimal sensitivity of 1 CLL cell per 10,000 leukocytes (0.01% or 10^−4^).

The most recent iwCLL guidelines (2) recommend MRD assessment with standardized methods in CLL patients enrolled in clinical trials aiming at obtaining a deep response. Since some drugs preferentially kill CLL cells in the PB showing a lower effect on the other compartments, the first step should be the MRD evaluation by FCM or other assays in the PB compartment, always expanding the analysis to the BM if PB MRD is negative. In agreement with previous reports (24, 35), we evaluated MRD by FCM in 225 paired PB and BM samples, proving a high degree of concordance (92.4%) (36). Among the PB MRD+/BM MRD+ (*n* = 117), significantly higher values of MRD were found in the BM compartment compared to PB. In the few discordant paired samples (*n* = 17), 94% were PB uMRD/BM MRD+ (36). Thus, in a CLL patient in the presence of a PB MRD positivity we can be certain that the respective BM sample will be positive. Contrariwise, the observation of a PB uMRD cannot exclude the presence of MRD in the BM that needs to be specifically analyzed (36). Given the multi-compartimental nature of CLL, residual cells can also be hidden in lymph nodes after therapy and could play a role in the emergence of a subsequent relapse. Although the standardized MRD testing methods rely only on CLL cells that circulate in the PB or BM, residual disease in the other compartments could be measured by evaluating plasma cell-free DNA (cf-DNA) using the most sensitive technologies now available, such as ddPCR and NGS-based approaches (see below).

### Flow Cytometry-Based Approach

FCM evaluation of residual CLL cells is mainly based on the differential expression of surface antigens between CLL and normal B cells. Historically, CD19/CD5 co-expression with demonstration of clonality by Ig light chain (κ or λ) restriction has been the simplest and most commonly used FCM approach for MRD evaluation, virtually applicable to all CLL cases but with a low sensitivity. The introduction over the years of an increasing number of antibodies in the work panel (see below) has allowed a more accurate definition of residual CLL cells. However, in order to save labor work, time, reagents and contain costs, the use of a simple upfront clonality assessment, by CD19/CD5/κ/λ screening, can identify samples with high levels of residual disease, in which a full multicolor MRD analysis is unnecessary (25).

The CD20 antigen has represented the best discriminator between normal and CLL B cells, as it shows a higher intensity of expression in the former. However, rituximab-containing treatments can mask the CD20 epitope with a downregulation of protein expression on the surface of normal B cells, making it difficult to dissect CLL cells from normal B cells. The introduction of CD79b to the CD19/CD5/CD20 combination resulted in a 2-log increase in terms of sensitivity compared to conventional 4-color analysis including CD19, CD5 and Ig light chain clonality restriction, improving the prediction of outcome in CLL patients treated with alemtuzumab (10, 11). In addition, the evaluation of CD43, an antigen homogeneously expressed on CLL cells (37–39), in combination with CD19/CD5/CD20 also appeared to be a useful approach. This 4-color combination allowed to achieve a 100% specificity in identifying residual CLL cells when compared to molecular biology techniques, up to a maximum sensitivity of 2.2 × 10^−4^ (40). Subsequently, the use of the CD81 antigen in combination with CD22/CD19/CD5, has allowed an accurate evaluation of MRD also in patients treated with rituximab-based therapies, with a good sensitivity and specificity (41).

Beside the choice of the antibody panel, another crucial aspect of FCM monitoring is the number of acquired events to reach the sensitivity of 1 leukemic cell in 10,000 normal cells (0.01% or 10^−4^).

Generally, for a correct MRD estimation, at least 500,000 events need to be acquired for each tube with the identification of at least 20 clustered events displaying a CLL immunophenotype. The presence of MRD is reported as the percentage of pathologic cells within the total leukocyte population. Conventionally, MRD is defined as undetectable if <0.01% or <10^−4^ (i.e., <1 CLL cell per 10,000 leukocytes), intermediate if <1% and ≥.01% or <10^−2^ and ≥ 10^−4^ (i.e., 1–99 CLL cells per 10,000 leukocytes) and positive if ≥1% or ≥10^−2^ (i.e., ≥100 CLL cells per 10,000 leukocytes).

The first consensus document by the European Research Initiative on CLL (ERIC) group has identified a standardized method for MRD identification and reporting, based on specific monoclonal antibody combinations and on codified fixed gating strategies, aimed at improving the sensitivity and reducing the inter-operator variability and false-positive rate (24). This assay is based on five 4-color antibody combinations: Igλ/Igκ/CD19/CD5; CD45/CD14/CD19/CD3; CD20/CD38/CD19/CD5; CD81/CD22/CD19/CD5; CD43/CD79b/CD19/CD5.

**Figure 1:**
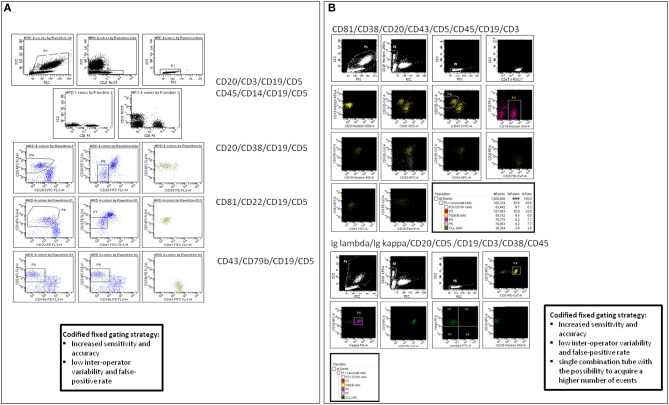
MRD analysis by flow cytometry (FCM). **(A)** ERIC standardized monoclonal antibodies combinations and analysis, according to Rawstron AC et al. **(B)** 8-colors panel designed in-house with a strategy of analysis based on the ERIC codified fixed gating.

The strength of this FCM panel is its applicability to all sample types and therapeutic regimens allowing an accurate separation of CLL cells from the background, with a high concordance with ASO IgH RQ-PCR for the detection of CLL cells above 0.01% (10^−4^). Moreover, this standardized 4-color FCM assay has proven highly reproducible, being validated in most multi-center randomized clinical trials (14, 42, 43). Our group performed a comparison between the in-house 4-color analysis (CD20/CD5/CD3/CD19; CD20/CD38/CD19/CD5; Igκ/Igλ/CD19/CD5) and the ERIC panel in 462 CLL samples, and identified slight differences in the amount of leukemic cells, with lower MRD values using the ERIC panel (Figure 1) (36). We concluded that the use of the CD81 and CD43 expression intensity evaluation, in the ERIC consensus method, allowed a more accurate identification of CLL cells. However, the ERIC assay can be challenging in poorly cellular samples, where it may be difficult to acquire an adequate number of cells.

The combination of more parameters in a unique tube has allowed to reduce the number of individual assessments required for each case. Accordingly, a 6-color FCM assay was developed in two tubes: CD3/CD38/CD5/CD19/CD79b/CD20; CD81/CD22/CD5/CD19/CD43/CD20 (25), increasing the possibility of acquiring up to 500,000 events or more even in post-treatment and often hypocellular samples. The possibility of combining all the required antibodies into a single tube thanks to the availability of 8- or 10-color FCM instruments, with the acquisition of a higher number of events per test can potentially improve the limit of detection below 10^−4^ (44, 45). The ERIC 8-color combination comprising CD19, CD20, CD5, CD43, CD79b, CD81, CD22 and CD3(26) allowed the reliable detection of residual CLL cells down to 0.001% (10^−5^) with a single-tube assay. Our group also demonstrated the applicability of a single 8-color combination tube (CD81/CD38/CD20/CD43/CD5/CD45/CD19/CD3) obtaining comparable MRD values with those resulting from the ERIC 4-color panel, bypassing the issue of sample cellularity that represents the main sensitivity limitation of FCM MRD analysis (36).

Additional markers such as CD200, CD23, ROR1 or CD160 can also be included (46–49).

Thus, in the last decade a standardization process has been progressively conducted in the field of FCM methodology and analysis for MRD evaluation in CLL, increasing FCM sensitivity and leading to comparable results across different laboratories and regardless of the type of treatment.

### PCR Techniques

In each patient, CLL cells are characterized by a unique IgH gene locus derived from the combination of variable (V), diversity (D) and joining (J) gene segments rearranged during early lymphoid differentiation. The unique *IGHV-IGHD-IGHJ* rearrangement of each leukemic B-cell clone can be amplified using consensus primers for *IGHV* and *IGHJ* genes at the 5′ and 3′ of the rearranged region (50). A monoclonal PCR product of the same size of that identified at diagnosis, usually with the presence of a polyclonal background referable to the normal B-cell population, allows the qualitative visualization of residual CLL cells.

MRD evaluation by RQ-PCR is based on the identification at diagnosis of a patient-specific molecular target on which sequence primers and probes are designed and then used to monitor the disease during clinical follow-up (51).

Different strategies for the design of primers and probes have been investigated (51–54). Briefly, one approach is based on the use of a single ASO primer in combination with a reverse consensus primer located at the 3' end of the *IGHJ* gene, with a fluorescent probe located between the two primers. The other approach combines both highly specific ASO primers together with probes; also the latter, in challenging cases, might be specific for the patient's rearrangement. It has been reported that the use of patient-specific primers and probes represents a more powerful strategy for RQ-PCR analysis in B-cell lymphoproliferative disorders, being independent of the mutational load of the *IGHV* and *IGHJ* regions, with evaluable results in more than 90% of cases (51). It is important to establish the sensitivity of each ASO primer used in the assay, as well as the conditions of amplification, on the diagnostic material that is serially diluted in normal mononuclear cells. On the basis of the dilution of leukemic cells, it is possible to quantify the MRD levels in the samples collected during/after treatment. The RQ-PCR approach using patient-specific primers and probes allows the identification up to 1 leukemic cell in 100,000 normal lymphoid cells (10^−5^) (55).

We have demonstrated in 185 FCM uMRD samples collected after chemoimmunotherapy that there is a sizable proportion of samples (22.7%) proving positive by ASO IgH RQ-PCR. Patients with FCM MRD-/RQ-PCR MRD+ showed a poor PFS similar to that of FCM MRD+ cases (36).

Challenges related to RQ-PCR-based MRD evaluations are represented by the need of diagnostic material for all MRD evaluations of each patient and the occurrence of the hypermutation process that requires a specific primer/probe design (see above).

Overall, RQ-PCR results in MRD monitoring of CLL are interpreted according to the guidelines developed for ALL by the European Study Group for MRD detection in ALL (ESG-MRD ALL) (34), today known as EuroMRD Consortium. These guidelines provided instructions on how MRD quantification should be conducted and proposed criteria for the unequivocal distinction of residual leukemic cells from the background, particularly for samples with a very low leukemic cells infiltration. Moreover, “positive, outside quantitative range” samples are also defined and identified as those in which the MRD quantification is impossible due to very low template copy numbers having a low tumor burden between the sensitivity and the quantitative range of the method, and for this defined as positive not-quantifiable (PNQ) samples (34) (see below). These concepts, developed for ALL, are also applied to CLL.

However, it has to be underlined that at the single patient's level the kinetics of MRD is more relevant than a single MRD assessment, since the increase of MRD over time and not only its persistence or “fluctuation” is eventually followed by clinical relapse.

### New Generation Approaches for MRD Monitoring: ddPCR and NGS

Despite the high sensitivity and specificity of RQ-PCR approach, as well as its established role for MRD evaluation in CLL, this method has important limitations especially due to the requirement of standard curves generated from dilutions of the tumor-specific target identified at diagnosis, and to the dynamic of intrinsic technical variations not fully eliminable. Moreover, a certain proportion of samples could fall in a window of inadequate quantification i.e., PNQ samples. To overcome these limitations, the ddPCR technique has recently been adopted for MRD measurement in different hematologic malignancies (31–33). This third generation quantitative method is based on a partition of the DNA template molecules into about 20,000 water-in-oil droplets, each representing a PCR reactor where a specific target is amplified. Droplets that contain the target gene are considered as positive by PCR amplification while those that do not are counted as negatives. At the end of the PCR process, it is possible to count the positive droplets (positive rate) with a direct and absolute quantification of the target with no need to compare with a reference or standard sample. Moreover, ddPCR is capable of detecting and quantifying molecular targets with high accuracy and precision especially when their concentration is very low, representing an ideal tool for MRD quantification (56–58). MRD measured by ddPCR in different hematologic disorders, such as multiple myeloma, mantle cell lymphoma, follicular lymphoma, ALL, has shown an excellent correlation with MRD measured by well-established RQ-PCR methodologies, with a high percentage of concordant results (31–33). No data have been so far reported in CLL. Our group has recently conducted a comparative study of ddPCR and RQ-PCR in more than 500 baseline and MRD samples from different lymphoid malignancies, including 116 CLL samples. In all disease entities investigated, a high correlation of the methods was found with most discordances recorded in samples with low RQ-PCR MRD levels, in which ddPCR was able to identify a quantifiable disease more reliably than RQ-PCR (59). Moreover, in a series of early stage FL from our group, ddPCR MRD was more significant than RQ-PCR MRD in predicting PFS after treatment (33). Thus, ddPCR may be considered as an alternative tool for MRD assessment in lymphoid malignancies, although its value needs to be conclusively documented in the context of prospective clinical trials. In the Euro MRD Consortium, ongoing efforts are being conducted for the inter-laboratory reproducibility of ddPCR MRD assessment and for the generation of standardized guidelines.

Also NGS technologies have been applied to monitor MRD. Different are the experiences in ALL and other hematologic diseases (60–63), while few data are available on the use of NGS in MRD monitoring in CLL (26, 29, 30). Logan et al (30). identified a good correlation between ASO-PCR and high throughput sequencing (HTS) with consensus V and J segment IGH primers in the MRD monitoring of 40 CLL patients after reduced intensity allogeneic SCT. In 16 of the 174 samples (9.2%) evaluated by both ASO-PCR and IGH-HTS and negative by ASO-PCR, a detectable disease was found by IGH-HTS (<10^−4^->10^−6^). This was highly predictive of 12-month disease-free survival compared to cases with a negative IGH-HTS MRD (<10^−6^) (37.5 vs. 93.3%; *p* = 0.0002) (30). Overall, the advantage of HTS approaches is the possibility to quantify MRD levels using consensus primers without the requirement of customized patient-specific primers, with a broader applicability than RQ-PCR methods and a sensitivity equally or higher than the more conventional quantitative approaches.

Moreover, NGS-based MRD assessment may not only overcome some disadvantages of PCR-based methods but also enable the analysis of the genetic diversity and clonogenic heterogeneity which may contribute to a better understanding of the biology of the disease. Thus, the definition of “monoclonality” of CLL could be challenged by the availability of genome-wide analyses (64). Since these methods are able to generate massive amounts of biologic information, an appropriate bioinformatic expertise for results interpretation and analysis is required. The applicability of NGS to MRD detection is not well-established. Work is in progress in this field within the EuroMRD Consortium.

The availability of HTS approaches has also expanded the sources of DNA for disease genotyping and monitoring. Plasma circulating tumor DNA (ctDNA) is readily detectable in patients with CLL, providing a unique opportunity for a comprehensive genotyping of the disease from all the involved compartments, including the lymph nodes, and for a non-invasive serial analysis of clonal evolution and possibly MRD investigation (65–67). In the study by Yeh et al. (68), the presence of ct-DNA as a measure of disease burden was compared to standard FCM MRD in the BM or PB in 30 time points and the results showed a complete concordance.

Other sources of nucleic acids, such as cell free-RNA/microRNA or those derived from exosomes could be explored in the future as biomarkers of residual disease in CLL, as done in other tumors (69, 70).

Ultra-deep NGS have shed light on the molecular mechanisms of the acquisition of resistance to a given treatment in CLL, as shown for *TP53* mutations under chemoimmunotherapy (71–73), *BTK* and *PLC*γ*2* mutations under ibrutinib (74–78) and *BCL2* mutations under venetoclax (79, 80). As a future prospective, MRD monitoring in CLL patients could drive the search of acquired mutations under a given therapy even months before the development of clinical relapse.

## Clinical Impact Of Mrd: From Chemoimmunotherapy To Novel Drugs

Up to 5 years ago, treatment of CLL relied on chemoimmunotherapy with FC or B or Chl combined with rituximab, chosen according to patients' age and comorbidities, and given as a fixed number of 28-days cycles, usually 6; cases with *TP53* deletion/mutation were excluded due to their refractoriness to chemoimmunotherapy and treated with steroids, alemtuzumab and SCT, if eligible. In the last 5 years, treatment approaches for CLL patients have been revolutionized by the introduction of novel agents such as anti-BTK ibrutinib and anti-BCL2 venetoclax, that have shown an unprecedented efficacy in CLL with *TP53* deletion/mutation and in R/R cases and that more recently have also moved to the first line setting, with some differences in the indications according to the country. In Europe, ibrutinib is indicated for the first-line treatment of CLL patients as single agent and in R/R patients as single agent or in combination with BR. Ibrutinib is administered indefinitely until progression or toxicity. Venetoclax is indicated as single agent for R/R CLL patients who failed chemoimmunotherapy and one BCR inhibitor, and in patients with *TP53* deletion/mutation who failed or are unsuitable for BCR inhibitors. Venetoclax+rituximab is now indicated in CLL patients who failed at least one therapy.

**Figure 2:**
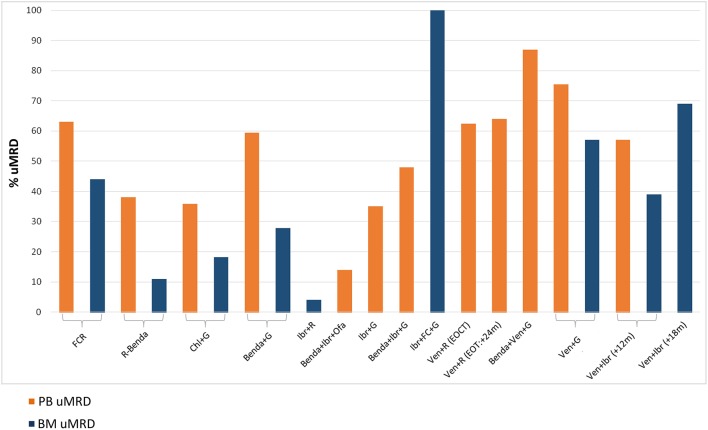
Percentages of undetectable MRD (uMRD) in peripheral blood (PB) and bone marrow (BM) compartments across different clinical trials. On the Left, the standard chemoimmunotherapy for CLL patients. On the Right, the most recent clinical trials incorporating BCR/BCL2 inhibitors. FCR, fludarabine+cyclophosphamide+rituximab (CLL8) (14, 81); R-Benda, rituximab+bendamustine (CLL10) (82); Chl+G, chlorambucil+obinutuzumab (CLL11) (83, 84); Benda+G, bendamustine+obinutuzumab (GREEN) (85); Ibr+R, ibrutinib+rituximab (Alliance A041202) (86); Benda+Ibr+Ofa, bendamustine+ibrutinib+ofatumumab (CLL2-BIO) (87); Ibr+G, ibrutinib+obinutuzumab (Illuminate PCYC-1130) (88); Benda+Ibr+G, bendamustine+ibrutinib+obinutuzumab (CLL2-BIG) (89); Ibr+FC+G, ibrutinib+fludarabine+cyclophosphamide+obinutuzumab (90); Ven+R (EOCT, End Of Combination Therapy) (EOT, End Of Therapy), venetoclax+rituximab (Murano) (23, 91); Benda+Ven+G, bendamustine+venetoclax+obinutuzumab (CLL2-BAG) (92); Ven+G, venetoclax+obinutuzumab (CLL14) (93); Ven+Ibr (+12 m, months), venetoclax+ibrutinib (TAP Clarity) (94); Ven+Ibr (+18 m, months), venetoclax+ibrutinib (95).

MRD data in CLL have largely derived from the chemoimmunotherapy era (Figure 2). In many countries, chemoimmunotherapy has today an always more limited role, given the recent indications of novel drugs in TN CLL patients. Chemoimmunotherapy could still be used in those patients whose biologic profile predicts the achievement of long-term uMRD. The clinical relevance of MRD in the novel drugs era needs to be further assessed.

### Chemoimmunotherapy

Numerous clinical trials have demonstrated that patients who achieve an uMRD following chemoimmunotherapy have a significant improvement in PFS and OS compared to those with persistent MRD (13–15, 81–83, 85, 96–98). MRD proved to be an independent predictor of outcome after chemoimmunotherapy, regardless of the achievement of CR or PR, type of therapy, other biologic prognostic factors and patients' characteristics before treatment (14, 15, 96–98). Thus, MRD resulted not merely a surrogate marker of biologically defined CLL subgroups. This has been extensively reviewed by Thompson and Wierda (99).

The ERIC 4-colors FCM MRD approach was successfully tested in two large prospective FCR-based clinical trials, with similar results: the German CLL Study group (GCLLSG) CLL8 trial, the first-line phase III study randomizing physically fit CLL patients to receive FC or FCR (81), in which MRD was prospectively assessed by 4-color FCM in 493/817 patients (14), and the MDACC experience in which 4-color FCM MRD was tested in all 237 TN CLL patients treated with FCR (15). Three MRD levels were identified: <10^−4^ (uMRD), ≥10^−4^ up to <10^−2^ (intermediate MRD), and ≥10^−2^ (high MRD). In the German trial, after FCR, PB MRD was undetectable in 63%, intermediate in 24% and high in 13% of patients, corresponding to a PFS of 68.7, 40.5 and 15.4 months, respectively, with a significant difference for all comparisons; PB uMRD and intermediate MRD showed a similar OS, significantly better than high MRD (14). FCR induced at the end-of-treatment (EOT) a BM uMRD in 44% of patients in the German trial (14) and in 43% in the MDACC trial (15). In both studies, uMRD correlated with a significantly longer PFS and OS, both in univariate and multivariate analyses, independently of treatment arm and genetic features. Moreover, MRD adds to the clinical response defined by conventional criteria, since patients in clinical CR or PR with uMRD showed the same outcome (15). This was confirmed in a large study including GCLLSG CLL8 and CLL10 trials, where patients with PB uMRD PR showed the same PFS and OS of PB uMRD CR and a better PFS than PB MRD+CR. In detail, patients with uMRD PR with residual splenomegaly showed the same good outcome of those with uMRD CRs, whilst cases with uMRD PR with lymphadenopathies had a worse PFS (96).

Finally, patients who achieved early BM uMRD status, after 3 courses of FCR, and who continued on treatment, did not reach an improvement in PFS compared to those who stopped treatment (15). Thus, this was the first suggestion on a possible MRD-based strategy to modulate the length of therapy i.e., to stop treatment with the achievement of BM uMRD, rather than to administer a defined number of treatment courses. An updated analysis of the MDACC series with a median follow-up of 57 months (100), showed that BM interim-MRD after the 3rd FCR cycle (C3) was >1% in 41.5% of patients and predicted a lower likelihood of uMRD status at EOT (9 vs. 64% for C3 MRD ≤1%, *p* < 0.001) and a shorter PFS (median 41 mo vs. 73 mo for C3 MRD ≤1%, *p* < 0.001). A yearly blood MRD monitoring performed in 85 patients with uMRD status at EOT, documented the conversion to MRD positive in 38, after a median of 48 mo after EOT. The “MRD relapse” anticipated the clinical progression by a median of 24 months, a potential window for early intervention strategies (100).

A multicenter Italian series of fit patients treated with FCR (101), showed that patients who had the longest PFS after FCR were those with mutated *IGHV* and a favorable FISH. In a small fraction (4/9) of low-risk patients alive and progression-free for 6 or more years from FCR (range, 6–9 years), FCM PB MRD was negative in all. In a long-term follow-up analyses of the above mentioned studies (14, 15), Fischer et al (102). showed that PB MRD by 4-color FCM at EOT was negative in 68% of *IGHV* mutated evaluated patients. Thompson et al (103) showed that 50.7% of *IGHV* mutated patients achieved uMRD by PCR at EOT; of these, PFS was 79.8% at 12.8 years follow-up. Moreover, 4-color FCM PB MRD at 12.8 years (9.5–14.7) was negative in all 15 long-term responders with mutated *IGHV*. Interestingly, MRD at the end of FCR has not the same significance in patients with unmutated vs. mutated *IGHV*, since unmutated *IGHV* CLL who achieved uMRD at the EOT showed a significantly shorter PFS than patients with uMRD and mutated *IGHV* (103). Moreover, patients with unmutated *IGHV* showed a more rapid re-emergence of MRD over time (median 42 months vs. not reached, *p* = 0.01) (103).

These observations proved the long-term persistence of a deep response among low-risk CLL patients after FCR; although representing a minority, this subgroup might still benefit from a conventional chemoimmunotherapy approach even in the era of novel compounds.

The phase III randomized trial CLL11 showed the superiority of 6-courses of obinutuzumab-Chl (G-Chl) on R-Chl or Chl single agent in 781 previously untreated and unfit CLL patients (83). MRD was evaluated by ASO-PCR, according to EuroMRD guidelines in the PB and in the BM only for patients in clinical CR. Results on MRD in the G-Chl vs. R-Chl arms, evaluated in the PB at the EOT in 474 patients and in the BM in 274, were recently updated with a median follow-up of 65.6 months (84). Overall, at EOT, 90 patients (19.0%) had PB uMRD (<0.01% or <10^−4^ i.e., <1 CLL cell per 10,000 leukocytes), 132 (27.8%) intermediate-MRD (<1% and ≥0.01% or <10^−2^ and ≥10^−4^ i.e., 1–99 CLL cells per 10,000 leukocytes) and 252 (53.2%) positive-MRD (≥1% or ≥10^−2^ i.e., ≥100 CLL cells per 10,000 leukocytes). Patients with PB uMRD had a median PFS of 56.4 months vs. 23.9 months for intermediate-MRD patients vs. 13.9 months for positive-MRD patients (*p* < 0.001 for all comparisons). Median OS was not reached (NR) in the PB undetectable and intermediate MRD categories vs. 60.0 months for PB positive-MRD patients (intermediate vs. undetectable, NS; positive vs. undetectable *p* < 0.001). Thus, MRD retains its prognostic significance even in elderly and less fit CLL patients treated with less intensive regimens. uMRD at EOT was significantly more common in patients receiving G-Chl vs. those receiving R-Chl (35.8 vs. 3.3% in the PB and 18.2 vs. 2.6% in the BM, respectively). At the multivariate analysis, PB MRD (positive vs. undetectable) at EOT independently predicted PFS—along with treatment arm (G-Chl vs. R-Chl), serum thymidine kinase and immunogenetic risk factors—and OS—along with Binet stage C, total CIRS score, serum thymidine kinase and immunogenetic risk factors (84). Unfortunately, the biologic profile of the elderly/unfit patients who achieved uMRD is unknown.

The FCR combination, the standard front-line therapy for fit CLL patients without del(17p), was compared in the randomized phase III study (CLL10) to the potentially less toxic combination consisting of BR (82), in previously untreated fit CLL patients without del(17p). 4-color FCM PB MRD was tested in 185/282 patients in the FCR arm and 170/279 patients in the BR arm (also in the BM for those who achieved a clinical CR). The achievement of a PB uMRD, significantly more frequent among FCR-treated CLL patients than in those receiving BR at the EOT (49 vs. 38% in the PB and 27 vs. 11% in the BM, respectively), translated into a longer PFS, confirming the results from the CLL8 study.

In the GREEN trial, a non-randomized, open-label phase IIIb study investigating G alone or plus chemotherapy (FC or Chl or B) in TN or R/R CLL patients, a subgroup analysis of 158 TN CLL patients receiving B+G was reported. ORR was 81.0%, with uMRD in 59.5% and 27.8% of patients for PB and BM, respectively (85). Interestingly, all patients in stage A and with mutated *IGHV* achieved uMRD, but also a high proportion of those with lymphocyte count ≥50 × 10^9^/l, disease bulk ≥5 cm, Binet stage B+C or unmutated *IGHV* did. Lower uMRD rates were seen in patients harboring 17p or 11q deletions.

These clear evidences on the association between MRD status and PFS prompted the application of a meta-regression model to the three German phase III trials (CLL8, CLL11, and CLL10), in order to determine whether the chemoimmunotherapy effect on MRD response in PB samples at the EOT can predict the effect of treatment on PFS (98). This model indicated a strong association between MRD and PFS, confirming the role of MRD as a surrogate primary end point in randomized CLL clinical trials.

### The era of BCR and BCL2 Inhibitors

#### Ibrutinib

The BTK inhibitor ibrutinib as single agent allows a long-term disease control that improves over time with its continuous administration in the majority of patients, even in those with *TP53* deletion/mutation, and confers an advantage in PSF and OS over ofatumumab in R/R CLL patients and over Chl in elderly TN (104–109), despite the achievement of a clinical CR only in a minority of cases. Extended follow-up of CLL patients treated with ibrutinib in the front line and R/R settings (106, 107, 109), showed an increase of CR rate over time beyond the first 12 months of therapy, thus ibrutinib single agent should be administered until PD or unacceptable toxicity in patients in which the achievement of a MRD negativity is not the treatment goal. CLL patients with unmutated *IGHV* seem to achieve over time a better clearance of the disease measured by FCM in the PB than mutated *IGHV*, although this difference is not significant in the BM (110).

Ibrutinib has been combined to anti-CD20 monoclonal antibodies in order to increase the quality of the responses. Results of 3 randomized multicenter clinical trials comparing the combination of ibrutinib (given continuously until PD) plus an anti-CD20 monoclonal antibody to chemoimmunotherapy in the first-line treatment of CLL patients were recently presented. Ibrutinib+rituximab vs. ibrutinib single agent vs. BR in TN elderly CLL patients (Alliance North American Intergroup Study A041202) (86); ibrutinib+rituximab vs. FCR in TN CLL patients with <70 years of age and no *TP53* deletion/mutation (ECOG-ACRIN Cancer Research Group, E1912) (111); ibrutinib+G vs. G-Chl in TN CLL patients either elderly >65 years of age or young at high risk for unfitness or genetics (Illuminate PCYC-1130) (88). In all the 3 trials, PFS was superior in the ibrutinib+anti-CD20 antibody arm than in the chemoimmunotherapy arm, with an advantage in OS compared to FCR arm in the E1912 study. The advantage was evident also in high-risk genetic subgroups with the exception of mutated *IGHV* CLL in the E1912 trial where ibrutinib+rituximab was equivalent to FCR. Regarding MRD, ibrutinib+G induced higher PB uMRD than G-Chl (35 vs. 25%) in the iLLUMINATE trial (88). Contrariwise, ibrutinib in combination with rituximab induced few uMRD (4% ibrutinib+rituximab vs. 1% ibrutinib single agent vs. 8% BR) in the A041202 trial, where the addition of rituximab to ibrutinib showed no advantage in ORR, PFS or OS compared to ibrutinib alone (86).

This was confirmed in a recent single center randomized trial including mostly R/R patients (*n* = 181) with few high-risk TN CLL with *TP53* deletion/mutation (*n* = 27), where the addition of rituximab to ibrutinib therapy did not improve the 3y-PFS (86.9 vs. 86%) or the 3y-OS (89 vs. 92%) compared to ibrutinib single agent and did not significantly increase the ORR (92% in both arms) or CR rate (26 vs. 20.2%) as best responses, despite the faster achievement of CR and lower level of BM MRD evaluated by 4-color FCM (112). However, only 6 patients became MRD negative, 5 in the ibrutinib+rituximab e 1 in the ibrutinib arm. A higher CR rate was noted in the ibrutinib+rituximab arm in patients with del17p, and especially in TN patients, in whom the addition of rituximab increased the CR rate from 20 to 50%, interestingly half of them with uMRD. However, due to the relatively small number of patients in these subgroups, none of these differences reached statistical significance (112).

Another concept is the administration of ibrutinib in combination with agents with different mechanisms of action, in order to achieve uMRD and discontinue the treatment. A limited duration of therapy (i.e., prefixed or up to uMRD) could avoid the continuous exposure to the drug, the development of mutations conferring resistance, cumulative toxicities and unaffordable costs. The GCLLSG CLL2-XXX phase II trials (87), enrolling both TN or R/R CLL patients, were designed with this purpose. They included 2 cycles of B as debulking phase in patients with high tumor burden disease, followed by an induction and maintenance with ibrutinib and obinutuzumab (CLL2-BIG)(89) or ibrutinib and ofatumumab (CLL2-BIO) or venetoclax and obinutuzumab (CLL2-BAG) (92). Therapy was continued for 24 months or until PD or excessive toxicity or confirmed uMRD in the PB by 4-color FCM (uMRD <10^−4^; intermediate-MRD ≥10^−4^- <10^−2^; high-MRD ≥10^−2^).

Although the rate of ORR was comparable among the 3 combinations, ibrutinib in combination with G induced higher PB uMRD than ibrutinib+ofatumumab at the end of induction (EOI) (48 vs. 14%), but the best combination resulted venetoclax+G (87% PB uMRD, see below) (92). These differences were magnified in the subgroup of patients with *TP53* deletion/mutation (ORR 81% in ibr+ofa, 100% ibrutinib+G, 94% venetoclax+G and PB uMRD at EOI 0, 12.5, 76%, respectively). Again, ibrutinib with anti-CD20 monoclonal antibodies does not seem the best agent to achieve uMRD in most patients in order to plan a MRD-based discontinuation.

Other combinations include ibrutinib and chemoimmunotherapy. A former experience, the HELIOS trial (113), comparing ibrutinib+BR vs. placebo+BR in 578 R/R CLL (with no del17p), showed that the continuous administration of ibrutinib after BR could contribute to disease clearance: ORR 83 vs. 68%, CR 10 vs. 3%, 18 m-PFS 79 vs. 24%. BM MRD was evaluated by 8-color FCM in patients with clinical CR/CRi and resulted undetectable in the ibrutinib-based arm more frequently than in the placebo-arm, although still in a minority of cases (13 vs. 5% in the intention to treat population) (113). The trial results have been updated: the 36-month PFS rates were 68.0 vs. 13.9%, and 36-m OS 81.6 vs. 72.9%, respectively. CR rates increased over time in the ibrutinib arm. uMRD response rates were 26.3% for ibrutinib+BR and 6.2% for placebo+BR (*p* < 0.0001) (114).

At the MDACC, a phase II trial combined ibrutinib+3 cycles of FC+obinutuzumab (iFCG) for the treatment of 45 TN CLL patients with mutated *IGHV*, in order to maximize the quality of response, to reduce the number of chemotherapy cycles and to stop ibrutinib in the case of uMRD (90). After the 3 FC cycles, the ORR was 100% with 39% CR/CRi and BM uMRD 89%; at +12 months CR/CRi were 81% and BM uMRD 100%, with 32 patients who could discontinue ibrutinib (90).

#### Venetoclax

The BCL2 inhibitor venetoclax in the first phase 1 dose-escalation study in R/R CLL patients induced an ORR 79% with 20% CR (22). MRD status was evaluated by FCM on BM samples in 17 of the 23 patients in CR, with 6 patients (35%) resulting MRD negative. However, none of the PR was evaluated for MRD, thus potentially underestimating the MRD negative rate (96).

In a recent report on the prolonged follow-up of R/R patients treated with venetoclax from 4 clinical trials, with only a minority receiving venetoclax combined with rituximab (VR), it has been shown that uMRD in the PB and BM was achieved in 27 and 16% of cases, respectively, mostly within 2 years of treatment (115). Both pretreatment factors (i.e., *TP53* deletion/mutation and *NOTCH1* mutation; refractoriness to BCR inhibitors; bulky lymphnodes) and depth of response (i.e., PB uMRD at 24 months) were independently associated with the duration of response to venetoclax (115).

Subsequently, VR was compared to BR in a randomized phase III trial in 389 R/R CLL patients (MURANO trial) (23). VR was given for 6 months [end of combination treatment, EOCT] and followed by venetoclax monotherapy for a fixed duration of up to 24 months [EOT]. Results in favor of VR were impressive: MRD negativity on PB samples at m+9 (EOCT) was achieved in 62.4 vs. 13.4%, on BM samples 27.3 vs. 1.5%; a 2y-PFS of 84.9 vs. 36.3% and 2y-OS 91.9 vs. 86.6% (23). The updated results of this trial were presented at the last ASH meeting and recently published (91). MRD in this trial was defined by a combination of ASO-PCR and FCM, that showed a concordance of 86%; concordance between PB and BM compartments in uMRD was 90%. With a median follow-up of 3 years, the advantage of VR on BR was confirmed: 3y-PFS 71.4 vs. 15.2%, with a median PFS NR vs. 17m and a 3y-OS 87.9 vs. 79.5%. Also, the quality and durability of the responses were confirmed. PB at m +24 [EOT, 130 pts] showed a uMRD4 (<10^−4^) in 64% and low-MRD (≥10^−4^- <10^−2^) in 18% of patients. Whilst 70% and 98% of patients with uMRD at EOT remained in uMRD with no PD, respectively, those with high-MRD (≥10^−2^) at EOT were a high risk subgroup. In fact, PD at 9.9 m after completion of venetoclax was 79% in patients with high-MRD at EOT, 13% in those with low-MRD and only 2.4% in uMRD patients. Different quantitative levels within the range of MRD positivity have been shown to be at least as important for PFS as the distinction between MRD negativity (below 10^−4^) and MRD positivity (≥10^−2^) in the chemoimmunotherapy setting (14, 84). It might be true even for venetoclax-based combinations (91), but this aspect is still unclear.

PB uMRD at EOCT predicted PFS independently of clinical response (CR or PR) and type of therapy. Residual nodal disease did not. Thus, VR followed by venetoclax monotherapy induced such deep remissions that became the first novel regimen allowing a fixed-duration treatment. Whilst patients with high-MRD are a subgroup at high risk of progression, patients with BM uMRD can maintain long-term responses after the discontinuation of venetoclax therapy post-VR (116, 117). Three patients (2 with MRD+ CR and 1 with MRD negative CR, although with CLL cells detectable below the threshold of 10^−4^) have been re-treated with venetoclax after an asymptomatic progression after 25, 29, and 43 months off, and re-attained a PR, followed in one patient by a PD 18 months later (117).

These results push in the direction of a MRD-driven duration of therapy with venetoclax, with safe interruption in patients with BM uMRD responses, and of a possible MRD-driven early re-introduction of venetoclax in cases who lose the response.

Results on the combination of venetoclax + G have been recently released and are impressive.

In the phase Ib trial by Flinn et al. (118), 46 R/R and 32 TN CLL patients received venetoclax + G for 6 months followed by continuous venetoclax until PD or toxicity (R/R) or for 1 year (TN). ORR was 95% with 37% CR/CRi for R/R patients and 100% ORR with 78% CR/CRi for TN patients. At month+3 after G discontinuation, PB uMRD by FCM was found in 64% and 91% of R/R and TN cases, respectively, BM uMRD in 62 and 78% of R/R and TN cases, respectively; 26% and 63% of patients were CR/CRi with BM uMRD. Among TN patients, 72% were found PB uMRD after venetoclax completion; 10 patients subsequently converted to a detectable MRD after about 6 months.

In the phase II CLL2-BAG trial (92), 31 R/R and 35 TN CLL patients were treated with an initial debulking phase with 2 cycles of B according to lymphocytosis, 6 cycles-induction with venetoclax + G, followed by a venetoclax + G maintenance up to 24 months. Treatment was discontinued in case of toxicity, PD or confirmed PB uMRD. ORR was 95% with 40% CR/CRi. PB uMRD by FCM was achieved at the EOI in 87% of patients (91% among the TN and 76% among those with *TP53* deletion/mutation), intermediate MRD in 8% and positive in 2%. Treatment was discontinued in about one third of patients under maintenance due to the PB uMRD achievement, with follow-up not yet available.

In the phase III CLL14 trial (93), impressive results were achieved in TN CLL patients with comorbidities. Patients received either venetoclax + G or Chl + G for a fixed duration of 12 months. Venetoclax + G resulted superior for the all the efficacy parameters and across all the biologic subgroups, although a detrimental impact of *TP53* deletion/mutation on the PFS is still present. In the venetoclax + G arm, ORR was 84.7% with 49.5% CR/CRi; PB and BM uMRD, assessed by ASO-PCR 3 months after therapy discontinuation were 75.5% and 56.9%, respectively, and were sustained. Patients with clinical CR and PB uMRD were 42.1% and those with clinical CR and BM uMRD were 33.8%.

The newest combination is represented by ibrutinib+venetoclax. The TAP CLARITY trial based on ibrutinib+venetoclax in 54 R/R CLL patients and with a MRD-driven stop therapy strategy, exceeded the primary endpoint to indicate promising efficacy. The ORR was 94%, CR/CRi 54%. At m +14, PB MRD4 was 57% (28/49) and BM MRD4 39% (19/49) (94).

The MDACC phase II trial employed venetoclax+ibrutinib in 80 TN CLL patients, with treatment discontinuation in those with BM uMRD at m +24. Responses improved over time: the CR rate was 88% at m +12 and 96% at m+ 18; BM MRD4 was 61% at m +12 (in 33 evaluated patients) and 69% at m +18 (95).

A phase I trial explored the combination of venetoclax, ibrutinib and G in 12 R/R patients, with the aim of maximizing the efficacy and try a fixed duration treatment, with promising results in terms of uMRD in both PB and BM (119).

Which is the most effective anti-CD20 monoclonal antibody in combination with venetoclax and whether the addition of an anti-CD20 monoclonal antibody to venetoclax+ibrutinib combination is needed remain two open questions (Figure 2).

Results from a number of first-line clinical trials are awaited. The UK NCRI FLAIR CLL10 trial for fit TN CLL with no del(17p) has been further extended to enroll 1516 CLL patients in order to compare FCR vs. ibrutinib+R vs. ibrutinib single agent vs. ibrutinib+venetoclax (duration of therapy defined by MRD). The GCLLSG CLL13 compares chemoimmunotherapy to venetoclax+R vs. venetoclax+G vs. venetoclax+ibrutinib+G in TN fit CLL patients without *TP53* deletion/mutation.

### Allogeneic Stem Cell Transplant and CAR-T Cells

In the last years, the role of SCT in high-risk CLL has been challenged by the efficacy of novel agents in CLL patients with *TP53* deletion/mutation and R/R disease, so much that the indications for SCT in CLL have dramatically changed (120). However, SCT in CLL is not abrogated and remains a curative option though for an increasingly limited and highly selected group of patients.

In the past years, the impact of MRD after SCT, especially after a reduced intensity conditioning, has been evaluated in retrospective and prospective studies based on conventional or highly sensitive methodology (18, 19, 30, 121, 122) and reviewed by Thompson and Wierda (99). Moreover, the correlation between *graft versus host disease* (GVHD), MRD and clinical response has contributed to demonstrate the existence and the protective effect of *graft versus leukemia* (GVL) against CLL (19, 121–123). In summary, a MRD-negative status 1 year after SCT can be achieved in about 50% of patients, independently of high risk genetics/mutations, predicts a long-term clinical remission and is durable, although rare late reconversions to MRD+ and subsequent clinical disease relapses can occur (19). The updated results of the CLL3X trial at 10-years showed that the absence of MRD at m +12 post-SCT was highly prognostic for a reduced relapse risk (10-year relapse 25 vs. 80% if MRD was present, *p* < 0.0001) (123). The protective effect of MRD negativity at m +12 was even more pronounced if MRD clearance occurred only after immunosuppression withdrawal, suggesting an effective GVL activity (10-year relapse 12%) (123). Because MRD monitoring results are available in real-time, a pre-emptive immune intervention in response to the results of MRD assessment after SCT is feasible and mandatory, such as tapering of immunosuppressive therapy, adoptive cellular therapies (i.e., donor-lymphocyte infusions) or even novel inhibitors (124). The analysis conducted in the GCLLSG CLL3X trial showed the advantage in terms of event-free survival (EFS) and OS in transplanted patients who underwent MRD monitoring respect to those who did not, according to the single center policy (19, 125), thus the CLL post-transplant setting is the only context where MRD monitoring is followed by a clinical intervention.

These concepts will be relevant in the era of CAR-T cell immunotherapy. Indeed, CAR-T cells cleared the BM from CLL cells in most of 24 treated cases, as documented by negative FCM in 17/21 (88%). Twelve of these patients underwent deep IGH sequencing, and 7 (58%) had no malignant IGH sequences detected in the BM. The achievement of molecular responses in CLL patients who responded by iwCLL criteria was associated with a 100% PFS and OS (median 6.6 months follow-up) after CAR-T cell immunotherapy (126–129).

## The Future of MRD in CLL

MRD is a powerful prognostic tool to predict the outcome of CLL patients in the context of several clinical trials and is now an accepted surrogate marker to assess treatment efficacy in randomized trials before clinical endpoints can be evaluated. Despite these evidences, no MRD-driven decision making in CLL patients is currently applied in the clinical practice according to the most recent iwCLL guidelines, with the only exception represented by the pre-emptive immunotherapy after SCT.

Moreover, although the achievement of uMRD in CLL is the desirable goal to maximize the length of PFS and the way to pursue the eradication of the disease, it should be kept in mind that this is not necessarily true for the entire CLL patients' population. In the era of several novel compounds that have changed the therapeutic scenario both in R/R and TN CLL patients, the achievement of a durable disease control rather than a MRD-negative remission with ibrutinib may be more desirable especially for older patients and/or for those with comorbidities, in order to spare toxicity. In all other patients, i.e., young, fit and/or high-risk patients, venetoclax-based combinations aiming at achieving BM uMRD are the most promising way to significantly impact on the subsequent relapse risk and to provide a long-term disease remission, if not cure.

Several are the potential clinical implications of MRD in guiding CLL patients' treatment, such that MRD is likely to move to the clinical practice in the near future for a personalized management of CLL patients. However, the validity of the MRD-driven approaches mentioned below is still an area of uncertainty that needs dedicated studies to be conclusively defined.

First, MRD could be useful to decide when to stop treatment. There are some evidences about the use of early uMRD to guide the reduction of the number of administered FCR cycles, an approach that could spare overtreatment/toxicity in FCR treated patients, nowadays limited to those with low-risk genetics. MRD as a tool to limit the duration of chemo-free combinations is under evaluation, with a relevant impact in terms of compliance, clonal selection/resistance, toxicity, and costs.

Second, in the near future, the longitudinal monitoring of MRD will also allow to study the kinetics of the disease: the increase/reappearance of MRD by itself could guide the re-challenge of therapy after treatment discontinuation.

Third, the subsequent screening of emerging mutations conferring resistance to a given drug (es. *BTK, PLCg2, BCL2* mutations), could guide the switching to a non cross-resistant agent or combinations of agents, in order to anticipate the clinical relapse.

Last but not least, it will be necessary to integrate MRD analysis with the biological characteristics of the leukemia, that is currently lacking in the most recent trials. In first place, time to clinical progression is determined by both residual tumor burden and the growth kinetics of any low-level residual tumor cells, in turn determined by the biological characteristics of the leukemic cells, as stated by Thompson (130). This will allow to identify biomarkers capable of recognizing upfront patients who will achieve uMRD with the newest combinations, in order to guide treatment choice in those CLL patients in which uMRD is the goal.

Implementation of novel methods to detect MRD with greater sensitivity, such as HTS may further improve the predictive accuracy of CLL MRD assessment.

Thus, the MRD measurement might become the most appropriate tool to assess efficacy and direct therapeutic decisions also in the clinical management of CLL, with a modulation of the type, quantity and duration of treatment based on the real need of each patient. In view of the increasing relevance of MRD assessment in the management of patients with CLL, the need of high-quality standardization and of referral laboratories within national/international networks dedicated to MRD analysis is paramount.

## Author Contributions

IG and SR wrote the manuscript and equally contributed. IS, MC, LN performed RQ-PCR. MP performed flow cytometry. LVC, LC, and CI performed ddPCR and NGS. AG and RF critically revised the manuscript.

### Conflict of Interest Statement

The authors declare that the research was conducted in the absence of any commercial or financial relationships that could be construed as a potential conflict of interest. The handling editor declared a shared membership of the Biological Studies Committee (CSB) of the Fondazione Italiana Linfomi, though no other collaboration, with one of the authors IG.
